# Minimal Symptoms From a 7‐Month Retained Guidewire Following Acute Non‐Tunneled Hemodialysis Catheter Insertion: A Case Report

**DOI:** 10.1002/ccr3.71281

**Published:** 2025-10-14

**Authors:** Mohammad Sadra Saghafi, Mahdiar Mahmoodi, Alireza Saghafi, Hossein Saghafi

**Affiliations:** ^1^ Student Research Committee Qom University of Medical Sciences Qom Iran; ^2^ School of Medicine Qom University of Medical Sciences Qom Iran; ^3^ Division of Nephrology, Department of Internal Medicine, School of Medicine Qom University of Medical Sciences Qom Iran

**Keywords:** central venous catheterization, foreign‐body migration, guidewire, guidewire retention

## Abstract

Central venous catheterization (CVC), commonly used for hemodialysis due to its rapid access and immediate utility, carries risks—including the rare complication of guidewire retention. While typically identified early, retained guidewires can occasionally go unnoticed and migrate, leading to delayed and unexpected symptoms. This case report describes an unusual instance where a retained guidewire, undetected for 7 months, presented as thigh swelling. The case highlights the importance of sustained clinical vigilance, even long after routine procedures. A 52‐year‐old man with early‐stage chronic kidney disease (CKD) underwent emergency hemodialysis via a non‐tunneled jugular catheter. Seven months later, he developed localized swelling near the right knee. Imaging revealed a fractured guidewire extending from the inferior vena cava (IVC) to the femoral vein. Before the scheduled surgical intervention, the guidewire spontaneously protruded through the skin and was self‐extracted by the patient. Follow‐up imaging showed two small residual fragments, but with symptom resolution, conservative management was chosen. At 6‐month follow‐up, the patient remained asymptomatic.

**Summary**

- Guidewire retention is a rare yet preventable complication of CVC, often overlooked due to delayed or absent symptoms.
- Though frequently associated with inexperience or high workload, it can occur even under skilled care.
- This case underscores the value of post‐procedural imaging review, direct supervision, and the potential role of AI in early detection to enhance patient safety.

**Abbreviations**
AI: artificial intelligence
CVC: central venous catheterization
ED: emergency department
ESKD: end‐stage kidney disease
IVC: inferior vena cava

## 1 Introduction

Hemodialysis procedures require reliable vascular access, commonly achieved through various types of catheterization methods such as arteriovenous fistulas, grafts, and tunneled and non‐tunneled central venous catheters [1, 2]. While CVC is widely used for hemodialysis and other critical interventions due to rapid insertion and immediate functionality, it carries recognized risks of complications [2, 3]. These complications include infection, vascular injury, hematoma formation, pneumothorax, cardiac arrhythmias, and, rarely, retention of the metallic guidewire used during insertion [3, 4, 5, 6, 7, 8, 9].

Guidewire retention is an uncommon but well‐documented complication of central venous catheterization, usually recognized promptly during or immediately following the procedure [2, 3, 8, 10]. However, in rare cases, retained guidewires can remain unnoticed, potentially migrating intravascularly over extended periods [11]. Such delayed discoveries often occur incidentally on imaging or when patients present with unusual symptoms resulting from the guidewire migration [11]. Previous reports have described retained guidewires causing vascular perforation, cardiac arrhythmias, thrombus formation, embolization to distant organs, or even external protrusion through the skin at distant anatomical locations [2, 3].

This report describes an exceptional and previously unreported case of minimal symptomatic presentation 7 months after inserting an acute non‐tunneled double‐lumen catheter via the internal jugular vein. The retained guidewire, initially unnoticed, migrated distally through the patient's venous system to the right thigh, where it manifested as localized swelling just above the knee. This rare presentation highlights the importance of clinical vigilance and thorough follow‐up evaluation after catheter placements, particularly in the presence of atypical symptoms, even long after the initial procedure.

## 2 Case History and Examination

A 52‐year‐old male with a history of prolonged hypertension, renal calculi, and early‐stage CKD for over a year, managed with losartan‐hydrochlorothiazide 50/12.5 mg twice daily, presented to the emergency department (ED) in late February 2024 with recurrent nausea and vomiting. Initial laboratory results are summarized in Tables 1 and 2.

**TABLE 1:** Initial hematology laboratory tests.

Test	Result	Unit	Reference range	Status
WBC	8.8	10^3^/μL	4–10	Normal
RBC	**3.24**	10^6^/μL	4.5–6.3	Low
Hb	**8.5**	mg/dL	12–16	Low
HCT	**26.5**	%	36–48	Low
MCV	81.8	FL	80–96	Normal
MCH	**26.2**	pg	27–31	Low
MCHC	32.1	g/dL	32–36	Normal
Platelets	207	10^3^/μL	140–450	Normal
ESR 1st hour	**88**	mm/h	Men: 0–22, Women: 0–29	High

*Note:* The bold values are representative of abnormality in measured laboratory tests.

**TABLE 2:** Initial biochemistry and coagulation laboratory tests.

Test	Result	Unit	Reference range	Status
PT	15.7	Seconds	11–15	High
PT control	13	Seconds	—	Normal
I.N.R	1.4	—	—	Normal
PTT	26	Seconds	20–45	Normal
Urea	**510.3**	mg/dL	10–50	Very high
Creatinine	**17.0**	mg/dL	0–1.4	Very high
Sodium	**122**	mEq/L	135–147	Low
Potassium	**5.6**	mEq/L	3.5–5.3	High
Blood sugar	90	mg/dL	60–200	Normal
Calcium	**7.2**	mg/dL	8.5–11	Low
C‐reactive protein	3.8	mg/L	< 10	Normal

*Note:* The bold values are representative of abnormality in measured laboratory tests.

Ultrasound revealed mild unilateral hydronephrosis along with multiple cysts in both kidneys, while a non‐contrast spiral CT scan showed no evidence of urinary stones. Given the severity of uremia and electrolyte imbalances, a non‐tunneled double‐lumen jugular venous catheter was inserted, and emergency hemodialysis was initiated. Hemodialysis was performed multiple times, and after 6 days, the patient was discharged with the diagnosis of end‐stage kidney disease (ESKD) and scheduled for routine dialysis. At the time of discharge, the non‐tunneled catheter remained in place to continue dialysis, and the patient was scheduled for arteriovenous fistula creation 1 week later. Following successful fistula maturation 3 months later, the catheter was removed and dialysis was continued via the arteriovenous fistula.

**FIGURE 1:**
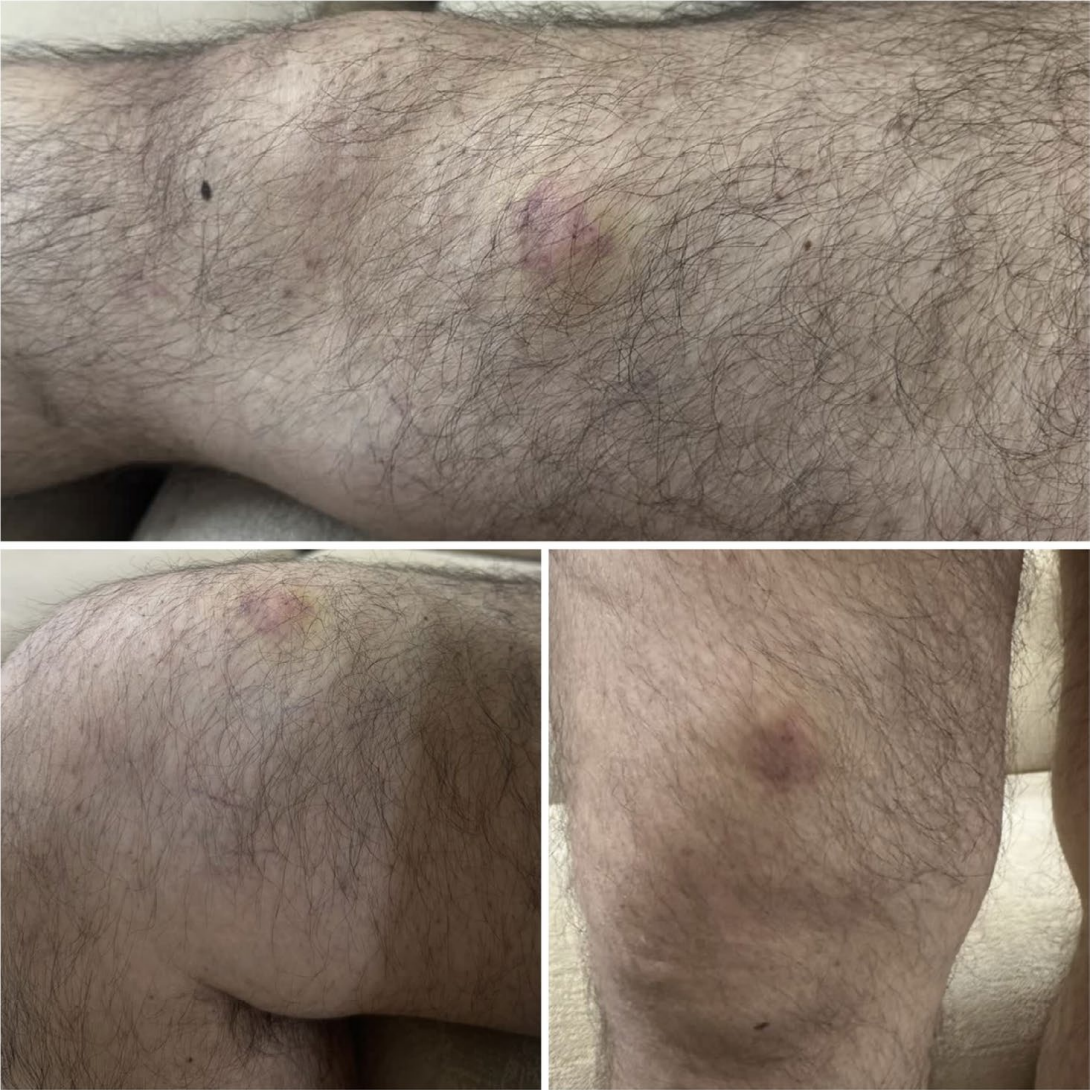
A localized swelling above the patient's right knee with mild erythema.

Seven months later, in early October 2024, the patient presented to the clinic complaining of focal swelling over the anteromedial surface of the right thigh, proximal to the knee. The swelling was approximately 2 × 2 cm, firm, resembling a lipoma, but associated with mild tenderness and erythema, as shown in Figure 1. Sonography suggested an inflammatory lesion, likely due to a linear foreign body.

**FIGURE 2:**
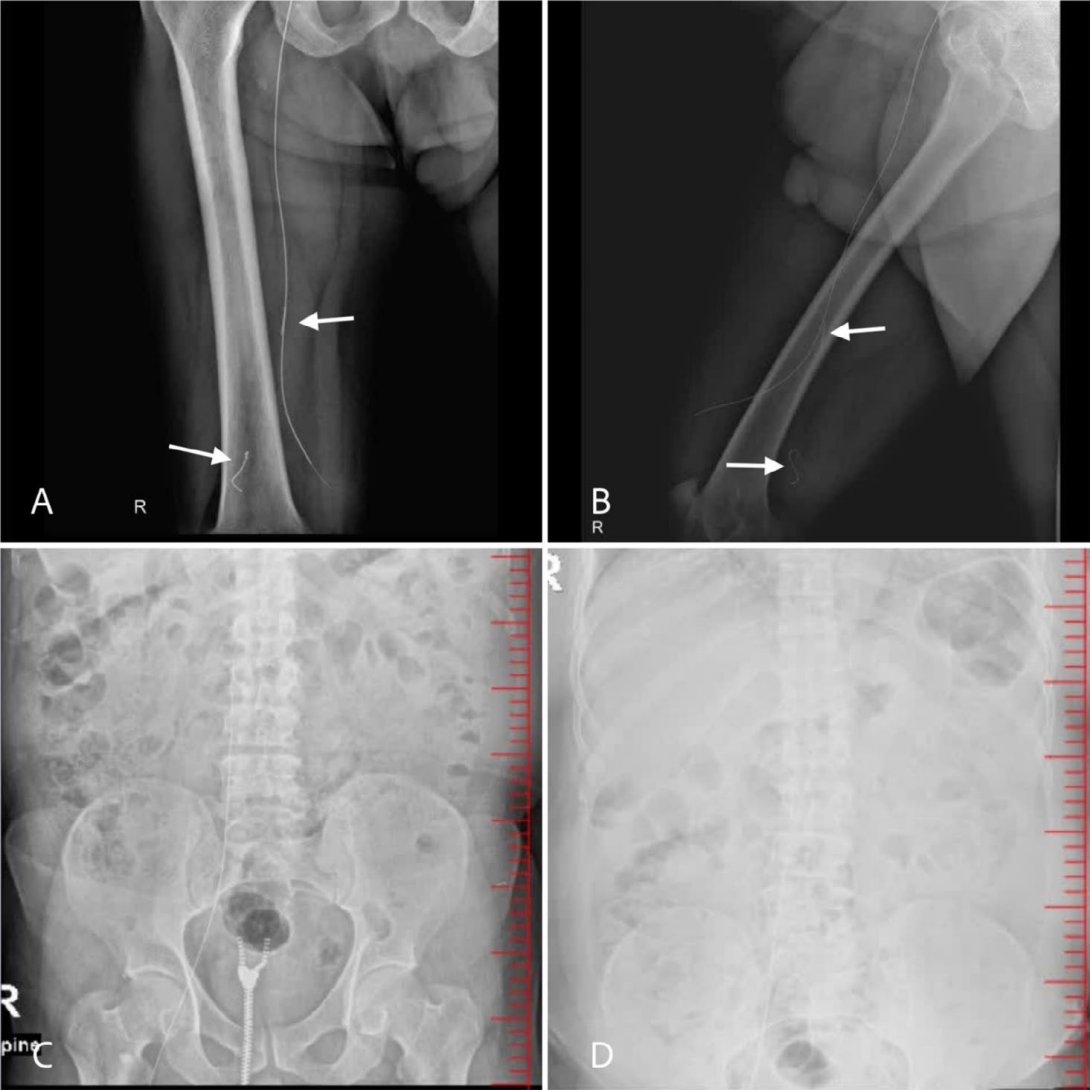
Femur and abdominal x‐rays revealing a guidewire extending from the inferior vena cava (IVC) to the femoral vein. (A, B) Anteroposterior and oblique views of the femur showing the distal end of the guidewire with two fractured segments (arrows). (C, D) Abdominal x‐rays showing the proximal end of the guidewire in the IVC.

To further investigate, femur and abdominal x‐rays revealed a retained guidewire extending from the IVC to the femoral vein, as demonstrated in Figure 2. The images also indicate that two segments of the guidewire were fractured and separated from the remaining portion. Given the patient's history of a non‐tunneled double‐lumen catheter insertion 7 months earlier, a diagnosis of retained guidewire was established. The patient was subsequently referred to a vascular surgeon for further management.

## 3 Outcome and Follow‐Up

**FIGURE 3:**
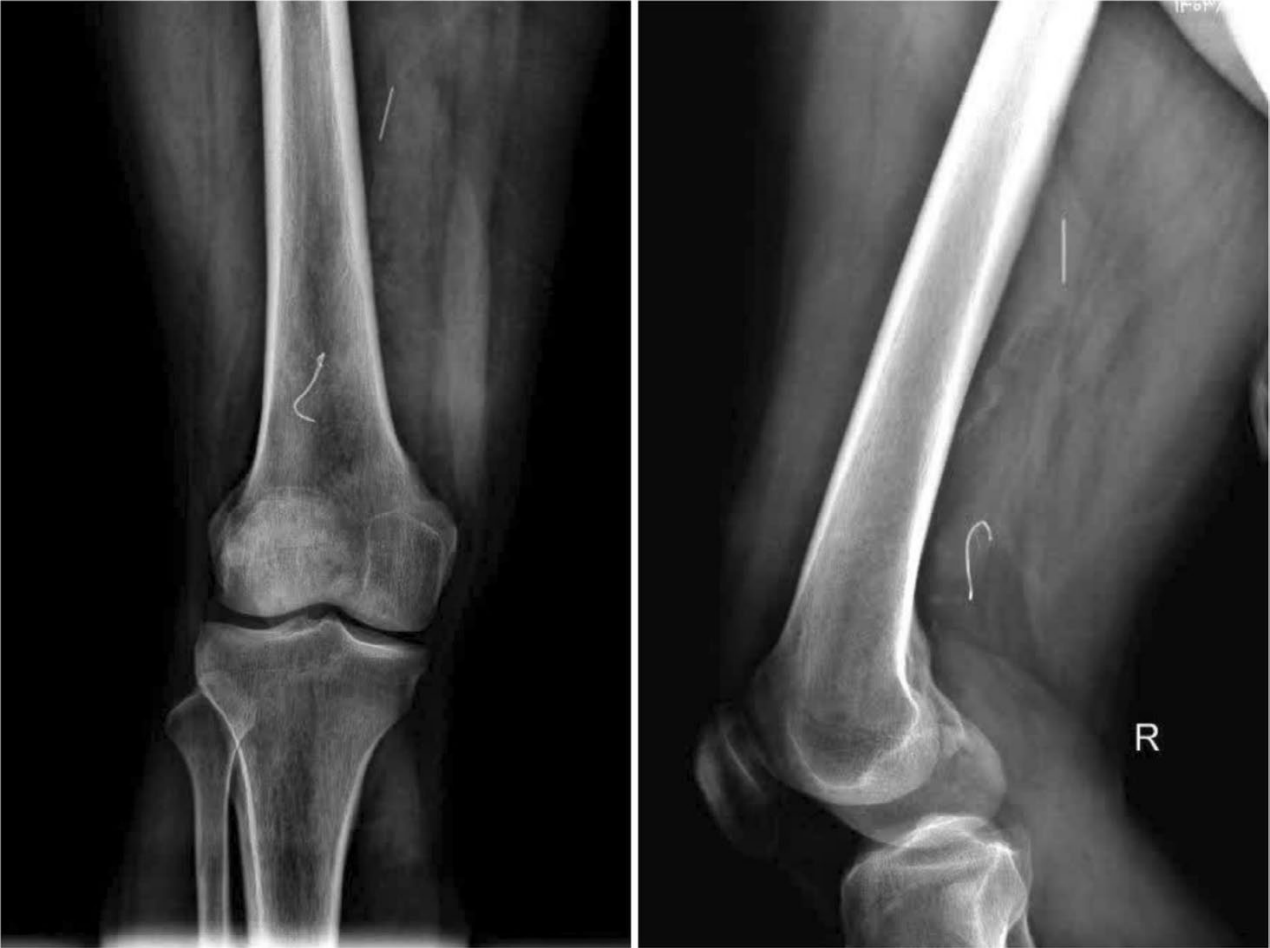
Anteroposterior and lateral views of the femur showing two small residual fragments after self‐removal of the guidewire.

Before the scheduled catheterization lab procedure by the vascular surgeon, the patient reported that the guidewire had spontaneously protruded through the skin at the swollen site, prompting him to extract it fully. Subsequent x‐ray imaging revealed two small residual fragments of the guidewire remaining in the femoral vein, likely corresponding to the previously noted fragment (Figure 3). Given the absence of significant symptoms and the resolution of the patient's clinical signs—including swelling, mild erythema, and tenderness in the right thigh—following the self‐removal of the majority of the guidewire, the vascular surgeon opted for conservative management with close follow‐up rather than pursuing further intervention to extract the retained fragment. The 6‐month follow‐up revealed an absence of symptoms and complications.

## 4 Discussion

CVC is a widely utilized clinical intervention that provides reliable vascular access for the infusion of intravenous fluids, administration of medications, and delivery of parenteral nutrition. It also serves as a critical conduit for performing hemodialysis and enables continuous monitoring of hemodynamic parameters [11], making it an essential component in the management of critically ill patients. Although CVC is generally considered safe when performed by experienced clinicians, it remains associated with a range of potential complications, including infectious, mechanical, and thromboembolic events [1, 11]. The frequency of these complications has increased, potentially as a result of higher clinical workloads or improved documentation and reporting of adverse events [2, 12]. Among these potential complications, guidewire retention is an infrequent but entirely preventable complication that remains a serious event, associated with a 20% mortality rate [13, 14].

Although it is a hazardous event, most of the time, a retained guidewire can go undetected for an extended period due to the absence of noticeable symptoms [14]. In cases where symptoms do arise, they may manifest as cardiac tamponade, abdominal discomfort, or chest pain [11, 15]. Alternatively, the retained guidewire may be discovered incidentally during imaging studies [14]. In this case, the patient exhibited pain and swelling in his right thigh. A previous case study described comparable pain and swelling where the guidewire protruded through the skin, leading the patient to remove it [1].

The increased incidence of retained guidewires following CVC has been linked to multiple factors, including procedural distractions, the clinician's experience level, high workload, and insufficient supervision, particularly in cases involving trainees. Notably, inadequate supervision is the most commonly reported risk factor in published cases [3]. However, in this instance, the procedure was carried out by a highly experienced anesthesiologist, highlighting that this complication can arise even in the absence of inexperience.

To minimize the risk of guidewire retention during CVC insertion, we propose the following recommendations:

1. Mandatory post‐insertion imaging: A post‐insertion x‐ray should be performed after CVC placement, including in emergency settings. Additionally, the x‐ray should be reviewed independently by both the physician and a nurse to ensure the absence of guidewire retention and verify the correct catheter position.
2. Integration of artificial intelligence (AI) in surveillance systems: Enhancing medical center surveillance systems through AI can significantly improve patient safety. Recent advances in AI, particularly in interpreting radiographic images, can automatically detect retained guidewires and alert healthcare providers promptly [16].
3. Direct supervision by an expert: When trainees perform the procedure, direct supervision by experienced clinicians is essential to ensure proper technique application and reduce the likelihood of guidewire retention [2].
4. Double checking the materials: Similar to the sponge count performed during surgeries, a post‐procedural double‐check of all used materials—conducted jointly by the operator and a nurse using a standardized checklist—could help identify any retained guidewire and prevent such complications.

**FIGURE 4:**
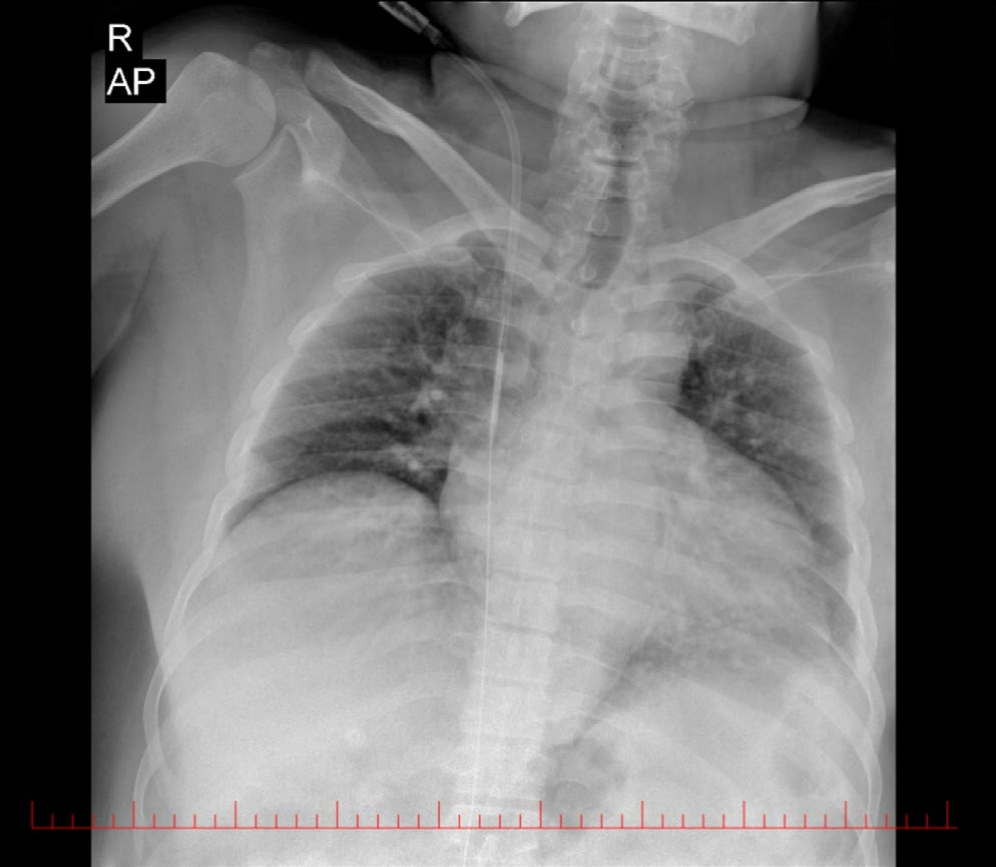
Post‐insertion chest x‐ray demonstrating the retained guidewire.

In this case, although the retained guidewire was visible on the post‐insertion x‐ray, as shown in Figure 4, the physician failed to detect it. This underscores the importance of implementing a secondary review process by another clinician or utilizing AI to detect retained guidewires. Factors such as work overload, inexperience, and fatigue—all of which are risk factors for guidewire retention—can also contribute to such oversights, emphasizing the need for additional safeguards to prevent these errors [3, 17, 18, 19, 20].

## 5 Conclusion

Human error remains an inherent aspect of medical practice, particularly in high‐pressure settings such as emergency care, where clinician fatigue, urgency, and procedural complexity can heighten the risk of oversight. While many of these errors may be benign, others can result in serious complications or present in atypical ways, as illustrated by this case. Such unexpected manifestations can lead to diagnostic confusion, unnecessary investigations, and added burdens on both the patient and the healthcare system. Therefore, implementing strategies to minimize preventable errors—such as routine post‐procedural imaging review, dual verification protocols, and the integration of AI into clinical workflows—should be considered essential steps toward enhancing patient safety and improving procedural reliability.

## Author Contributions

**Mohammad Sadra Saghafi:** conceptualization, data curation, investigation, visualization, writing – original draft, writing – review and editing. **Mahdiar Mahmoodi:** visualization, writing – original draft, writing – review and editing. **Alireza Saghafi:** methodology, validation, writing – review and editing. **Hossein Saghafi:** conceptualization, investigation, project administration, resources, supervision.

## Ethics Statement

This case report follows the ethical guidelines and is in line with Iran's domestic legal requirements.

## Consent

Written informed consent is obtained from the patient to publish this report in accordance with the journal's patient consent policy.

## Conflicts of Interest

The authors declare no conflicts of interest.

## Data Availability

The clinical data related to this case report are available on request from the corresponding author. The data are not publicly available due to privacy and ethical restrictions.
